# Image Motion Deblurring Based on Deep Residual Shrinkage and Generative Adversarial Networks

**DOI:** 10.1155/2022/5605846

**Published:** 2022-01-21

**Authors:** Wenbo Jiang, Anshun Liu

**Affiliations:** ^1^School of Electrical Engineering and Electronic Information, Xihua University, Chengdu 610039, China; ^2^Sichuan Provincial Key Laboratory of Signal and Information Processing, Xihua University, Chengdu 610039, China

## Abstract

A network structure (DRSN-GAN) is proposed for image motion deblurring that combines a deep residual shrinkage network (DRSN) with a generative adversarial network (GAN) to address the issues of poor noise immunity and low generalizability in deblurring algorithms based solely on GANs. First, an end-to-end approach is used to recover a clear image from a blurred image, without the need to estimate a blurring kernel. Next, a DRSN is used as the generator in a GAN to remove noise from the input image while learning residuals to improve robustness. The BN and ReLU layers in the DRSN were moved to the front of the convolution layer, making the network easier to train. Finally, deblurring performance was verified using the GoPro, Köhler, and Lai datasets. Experimental results showed that deblurred images were produced with more subjective visual effects and a higher objective evaluation, compared with algorithms such as MPRNet. Furthermore, image edge and texture restoration effects were improved along with image quality. Our model produced slightly higher PSNR and SSIM values than the latest MPRNet, as well as increased YOLO detection accuracy. The number of required parameters in the DRSN-GAN was also reduced by 21.89%.

## 1. Introduction

Image blurring can be caused by camera shaking or fast motion of a target object during exposure, which can be problematic in space exploration [1], facial recognition [2], video surveillance [3], and medical image recognition [4]. Blurring caused by relative motion between the camera and the target not only reduces the accuracy of human visual perception but also creates challenges for subsequent computer vision analysis. Thus, how to prevent image degradation from blurring and improve image quality at the same time has always been an urgent problem in the field of image processing.

Motion deblurring techniques can be divided into traditional and deep learning methods. Traditional deblurring often involves statistical prior modeling, while deep learning methods are generally driven by data. Conventional deblurring methods typically restore images by first estimating a motion blur kernel [5–14]. However, these approaches have limitations, such as complicated calculations, high noise levels, excessive blur kernel estimation requirements, heuristic parameter adjustments, and low generalizability, which often prevent their use in various scene types. As such, this paper focuses on current popular deep learning methods.

In recent years, the development of deep learning has led to an expanded use of convolutional neural networks (CNNs) for image processing. Xu et al. [15] first proposed a nonblind deblurring method based on CNNs. Subsequently, several algorithms utilizing deep learning to estimate blur kernels and traditional methods to restore images have since been proposed in recent years [16–18]. Sun et al. [16] proposed a method to estimate the blurred kernel using CNNs, but the algorithm assumes that the image is locally uniform blurred. Chakrabarti et al. [17] proposed a method to estimate the blurred kernel which uses filters to obtain image features and combined with neural networks. This method can estimate blurred kernels of different sizes, but the edge feature will be blurred in the restored image. Gong et al. [18] used a fully CNN to directly estimate the motion flow of a blurred image. However, these techniques also suffer from ring artifacts as blur kernel estimation accuracy is often insufficient, which can cause blurring to be nonuniform across an image. Nah et al. [19] first proposed an end-to-end deep learning method to recover images in dynamic scenes. However, this algorithm mainly relies on the high-capacity network model, and the network structure design does not do a good job of considering the properties of the blurred process. Kupyn et al. [20] proposed a new method of image deblurring based on a generative adversarial network (DeblurGAN). This algorithm uses the residual network as the core block of GAN and uses target detection as the evaluation method. However, this algorithm suffers from mediocre performance in objective evaluation. Kupyn et al. proposed DeblurGAN-v2, a method that improved DeblurGAN by applying a pyramid network to GAN [21]. These two algorithms demonstrate that GAN models can better retain details and texture information in images without the need to estimate a blur kernel. Although Suin et al. [22] and Zamir et al. [23] achieved better performance, these models required larger parameter quantities. In addition, neither algorithm considered noise in the sample images. In the past 20 years, soft thresholding has often been used as a key step in signal denoising [24,25]. However, identifying the optimal threshold can be difficult with conventional algorithms, as it may vary from case to case.

As such, this paper focuses on end-to-end GAN models and the processing of noise for image deblurring. In summary, image motion deblurring based on deep learning offers the advantages of low dependence on manual experience, fast recovery speed, and strong generalizability. However, this approach requires significant amounts of supporting data. Existing deep learning algorithms also suffer from issues, such as impractical restoration steps, complex network models, and poor robustness.

To address these issues, this paper proposes an image motion deblurring process based on a DRSN and a GAN, which modifies the structure of the GAN in several key ways. The primary contributions of this paper are as follows:

1. First, an end-to-end approach was applied, which avoids ringing effects and simplifies algorithm steps.
2. Second, a DRSN was used as the generator in a GAN to remove noise from input images while learning residuals and improving generalization performance. The internal structure of the DRSN was adjusted by moving the BN and ReLU layers to the front of the convolution layer, making the network easier to train.
3. Finally, deblurring effects and generalizability were verified using the GoPro, Köhler, and Lai datasets.

Experimental results showed the proposed DRSN-GAN algorithm yielded the best restoration performance compared with similar deep network structures, such as DeblurGAN and DeblurGAN-v2. It also achieved the best generalizability compared with the state-of-the-art algorithms, such as Deep Deblur, SRN, DeblurGAN, DeblurGAN-v2, the method of Suin et al., and MPRNet. Therefore, the DRSN-GAN image restoration method proposed in this paper represents an improved image restoration algorithm.

The remainder of this paper is organized as follows.  Section 2 introduces image motion deblurring.  Section 3 provides a brief overview of the classical GAN and a detailed elaboration of the developed DRSN-GAN.  Section 4 discusses the loss function. Experimental comparisons are given in Sections 5 and 6 conclude the paper.

## 2. Image Motion Deblurring

Image motion deblurring is used to recover a clear image from a blurred image. Most existing algorithms are based on the following blur model:

(1) $$\begin{array}{c} I_{B}=k*I_{S}+N, \end{array}$$

where *I*_*B*_ is a blurred image, *k* is a point spread function (PSF) defining the blur kernel, *I*_*S*_ is a clear image, ^*∗*^ represents a convolution operator, and *N* is an additive noise term. The primary goal of image deblurring is to restore *I*_*B*_ to a representation as close to *I*_*S*_ as possible. If *k* is known during deblurring, the process is called nonblind deblurring. Otherwise, it is referred to as blind deblurring. In practical scenarios, the blurring kernel *k* is typically unknown or difficult to determine. As such, this study focuses on the common case of an unknown blur kernel.

Conventional blind deblurring methods [5–14] first estimate the blur kernel and then deconvolve the blurred image with the estimated blur kernel to obtain a clear image. This kind of methods rely heavily on the accurate estimation of blurring kernels and are only suitable for specific types of blurring, which complicates the process for practical scenes. However, the development of deep learning and end-to-end techniques [16–28] has created new solutions for blind deblurring problems. End-to-end models offer several advantages, including the avoidance of blur kernel estimation, simple algorithm steps, and lower calculation costs. In this study, a GAN (with an ability to retain image details [20]) and a depth residual shrinkage network (with an ability to process noise) are combined and applied to image motion deblurring for the first time.

## 3. Generative Adversarial and Deep Residual Shrinkage Networks

### 3.1. Classic GANs

GANs are a type of generative deep learning model, first proposed by Goodfellow et al. [29] in 2014. GANs have achieved remarkable success for image superresolution reconstruction [30], image translation [31], style transfer [32], mutual generation of text images [33], and image in-painting [34, 35]. In addition, this model can in theory approach any probability distribution. The primary optimization goal of GANs is to achieve Nash equilibrium [36], with an objective function defined as follows [29]:

(2) $$\begin{array}{c} \underset{G}{\min}\underset{D}{\max}V\left(D,G\right)=\underset{x∼P_{r}}{E}\left[\log\left(D\left(x\right)\right)\right]+\underset{\tilde{x}∼P_{g}}{E}\left[\log\left(1-D\left(\tilde{x}\right)\right)\right], \end{array}$$

where *G* is the generator, *D* is the discriminator, *E* is the expectation, *x* is sampled in the real data distribution *P*_*r*_, $\tilde{x}$ is sampled in the model-generated data distribution *P*_*g*_, and the input is a simple noise distribution.

The classic GAN is immature and exhibits issues such as a vanishing gradient and mode collapse problems that have limited its development. As a result, several GAN-based variants have been proposed to overcome these issues, including conditional GAN (CGAN) [37], deep convolutional GAN (DCGAN) [38], Wasserstein GAN (WGAN) [39], least squares GAN (LSGAN) [40], and BigGAN [41]. Their mechanisms, advantages, disadvantages, and applicable scenarios are provided in Table 1.

### 3.2. Improved GANs

The comparative analysis in Table 1 demonstrates that WGAN solves the problems of unstable training and model collapse in the original GAN. WGAN replaces the KL and JS divergence in conventional GAN with the Wasserstein distance, making the gradient smoother. As such, the model proposed in this paper was developed using a WGAN framework.

**Figure 1:**
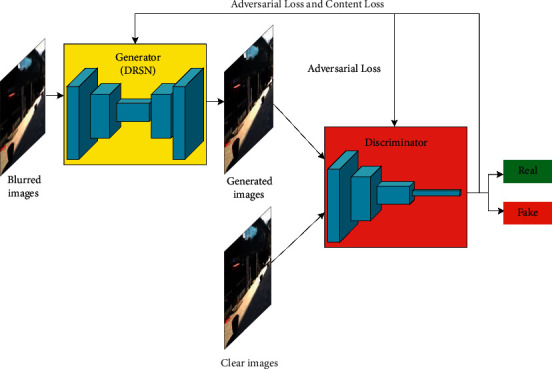
Improved GAN model.

**Figure 2:**
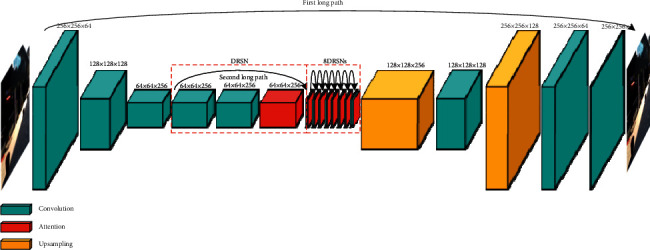
A structural diagram of the generator network.

**Figure 3:**
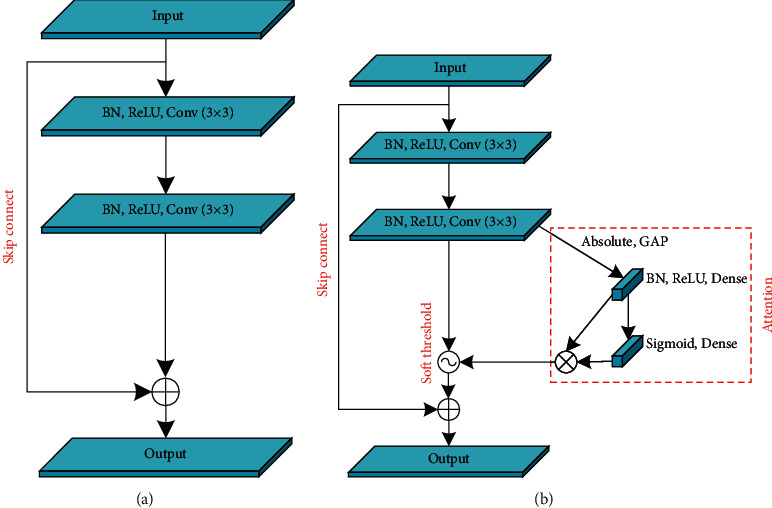
Comparison of two deep residual network structures.

The improved motion blur removal model is shown in Figure 1, in which the yellow box represents our contributions. A DRSN was used as the backbone network, allowing the generator to remove noise while learning residuals. The specific network structure is shown for the generator in Figure 2 and for the DRSN in Figure 3. The purpose of the generator is to learn potential distributions of image data samples, while the function of the discriminator is to determine whether input samples are real or produced by the generator. The image input to the discriminator was produced by the generator and its corresponding clear image in the data set. During training with adversarial and content loss, the abilities of the generator and discriminator are constantly improved until the entire network reaches a Nash equilibrium state. When the discriminator cannot identify the source of data, it can be approximately considered that the generator has learned the distribution of real image data.

In this study, the quality of images generated by the GAN was increased by making improvements to the generator module. Nine deep residual shrinkage networks were used as the GAN generator module. Prior studies have used DeblurGAN for image motion deblurring, combining a residual network and a GAN. However, this approach does not consider noise in the image, which affects restoration quality, model training rates, and convergence speed. Thus, in this study, the residual network was replaced by a DRSN, in order to solve the problems caused by DeblurGAN.

#### 3.2.1. Improved Generator Network Structure

The purpose of the generator is to reconstruct clear images from blurred input images. Therefore, it must not only retain structural details in the input image but also eliminate blur and noise to the extent possible. Unlike in the standard GAN, input to the generator *G* used in this paper was not random noise, but a blurred image to be restored.

The generator network structure shown in Figure 2 consists of four convolutional layers, nine DRSNs, and two transposed convolutional layers. The structure of layers one and four are the same (64 and 3 filters, resp., a kernel size of 7, a stride of 1, and a ReflectionPad buffer). The structure of convolutional layers two and three are also the same (256 and 512 filters, resp., a kernel size of 3, a stride of 2, and the “same” padding). A DRSN consists of BatchNorm (batch size of 1), ReLU, 3 × 3 Conv (256 filters, a kernel size of 3, a stride of 1, and a ReflectionPad buffer), dropout (rate of 0.5), identity connection, and attention module. In Figure 2, only one DRSN is shown in its entirety, while the remaining 8 DRSNs are simplified for illustration purposes.

First, the input blurred image was convolved with one 7 × 7 and two 3 × 3 convolution kernels in the convolution layer to ensure that the generating network can extract fuzzy image features from the pixel level to the content level during learning. Extracted shallow features were then input to a 9-deep residual shrinkage network used to acquire deep features [42]. Deep features were deconvolved to ensure the generated and input images were of the same size in the generation network. Deconvolutions were replaced by upsampling and convolution steps to avoid the chessboard effect. In addition, input to the first layer was directly transferred to the last layer through a global connection. As a result, the network only needed to correct residual errors, which increased training and convergence rates. This generator network structure is similar to a DeblurGAN generator, as 9-deep residual networks were modified to produce 9-deep residual shrinkage networks. This process is represented by the red box in Figure 2.

#### 3.2.2. Deep Residual Shrinkage Network Structure

Deep residual shrinkage networks (DRSNs), an improved version of deep residual networks (ResNets) [43], formed the core block of a generator combining deep residual networks, attention mechanisms [44], and a soft threshold function. A conventional deep residual network is shown in Figure 3(a). The quality of learned features and the resulting deblurred images can suffer when significant noise is present in the input images. In this study, an improved structure is developed that is less sensitive to noise, represented by the red dashed box in Figure 3(b). The sequence of the convolutional, batch normalization, and ReLU activation function layers is then adjusted. Specifically, the batch normalization layer and the ReLU activation function layer were positioned before the convolutional layer, making the network easier to train [45].

This architecture improved the ability of the deep neural network to extract useful features from noisy signals. In this algorithm, a small fully connected network was added before the output of the classic deep residual network. The working principle involves identifying unimportant features, using an attention mechanism and setting them to zero with a soft threshold function defined as follows [42]:

(3) $$\begin{array}{c} y=\left\{\begin{array}{c} x-\tau ,\;x>\tau ,\\ \text{0,}\;-\tau \leq x\leq \tau ,\\ x+\tau ,\;x<-\tau , \end{array}\right. \end{array}$$

where *x* is the input feature, *y* is the output feature, and *τ* is the threshold (a positive parameter). Instead of setting the negative features to zero in the ReLU activation function, soft thresholding sets the near-zero features to zero so that useful negative features can be preserved. The attention mechanism algorithm is detailed in Table 2 below [46].

#### 3.2.3. Discriminator Network Structure

**Figure 4:**
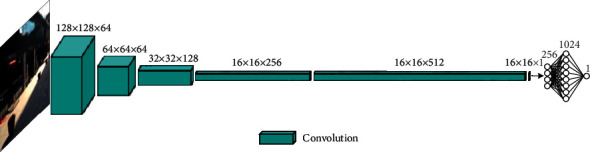
The discriminator network structure.

The discriminator network structure used to determine whether an input image is true or false is shown in Figure 4. Here, “true” indicates a real initial sample, while “false” indicates it was produced by the generator. The discriminator also assists the generator in producing a deblurred image closer to the real image. The input dimensions of the discriminator and the output dimensions of the generator are both 256 × 256 × 3. The network structure is primarily composed of convolutional, batch standardization, and activation module. A leaky ReLU (LReLU) with a parameter of 0.2 was used as the activation function, and a sigmoid function was connected behind the last layer of the discrimination network. The output mapped within [0, 1] represents the confidence level. The DRSN-GAN algorithm is outlined in detail in Table 3 below [46].

## 4. Loss Function

The choice of loss function directly determines the goals of deep learning and the effectiveness of a training model. The loss function used for image restoration tasks in this study was composed of two parts: adversarial and content loss. The purpose of adversarial loss is to restore textural details, while content loss restores more general content. Under the constraints of loss function, the generator and discriminator are trained against each other, and finally, they reach the Nash equilibrium state. The total loss function is defined as

(4) $$\begin{array}{c} L=L_{\text{GAN}}+\lambda \cdot L_{X}, \end{array}$$

where *L*_GAN_ denotes the adversarial loss, *L*_*X*_ is the content loss, and *λ* is the loss weight used to balance multiple loss functions. The value of *λ* was varied from 50 to 100 with an interval of 10. Experimental results showed the restored image exhibited better visual effects as each evaluation index was optimized for *λ* = 100. The following experiments were all conducted using these ideal conditions.

### 4.1. Adversarial Loss

Studies have shown that the introduction of an adversarial loss function can be used to reconstruct detailed texture information, making the generated image sharper and more visually acceptable. The rapid development of GANs in recent years has led to the emergence of several adversarial loss functions. Among these, common algorithms include square loss, cross-entropy loss, and Wasserstein distance loss functions. This study used Wasserstein distance, proposed by Arjovsky et al. [39], as it offers superior smoothing. This term is defined as follows:

(5) $$\begin{array}{c} L_{\text{GAN}}=\sum_{n=1}^{N}-D_{d}\left(G_{g}\left(I_{B}\left(n\right)\right)\right), \end{array}$$

where *N* is the batch size, *D*_*d*_ is the trained discriminator, *G*_*g*_ represents the trained generator, and *I*_*B*_(*n*) is the blurred input image from a batch.

### 4.2. Content Loss

Content loss functions restrict generated images for the purpose of semantic consistency with the input image to avoid distortion. A VGG19 network was employed due to its simplicity and practicality. It was pretrained on the ImageNet dataset [47] to acquire clearer images and improve perceptual effects. Content loss was calculated using feature differences between clear images and generated images extracted by the VGG19 network. The content loss function is defined as [48]

(6) $$\begin{array}{c} L_{X}=\frac{1}{W_{i,j}H_{i,j}}\overset{W_{i,j}}{\underset{x=1}{\sum }}\overset{H_{i,j}}{\underset{y=1}{\sum }}{\left(\phi_{i,j}{\left(I_{S}\right)}_{x,y}-\phi_{i,j}{\left(\bar{G}\left(I_{B}\right)\right)}_{x,y}\right)}^{2}, \end{array}$$

where Φ_*i,j*_ is the feature map acquired in the *j*th convolution (after activation but before the *i*th largest pooling layer) and the VGG19 network is trained in advance. The terms *W*_*i,j*_ and *H*_*i,j*_ represent the dimensions of the feature map.

## 5. Simulation Analysis

### 5.1. Experimental Settings

The GoPro dataset is the public database with the highest resolution, the largest scale, the most scenes and the most widely used in image deblurring research. This dataset provides realistic images by simulating the process of image blurring from real-time shooting. The data are comprised of 3214 pairs of blurred and sharp images with a resolution of 1280 × 720 × 3. The training set consists of 2103 pairs and the test set includes 1111 pairs. Images from the training set of GoPro dataset [19] were used as the training set, with a resolution converted from 1280 × 720 × 3 to 256 × 256 × 3 (to reduce training time). The Keras deep learning framework was used to implement the model developed as part of the study. The experimental platform was established in PyCharm, with a content weight loss *λ* set to 100 (based on empirical evidence). The batch size was set to 1, reflecting the task of individual image restoration. The adaptive moment estimation (ADAM) optimizer was adopted for training of the model, with a learning rate of 10^−4^. The remaining hyperparameters in the ADAM optimizer were set to default values in Keras. The discriminator was trained for five iterations and the generator was trained only once. This is because, prior to training the generator, the discriminator must be trained to a certain degree of discrimination. The network model was trained in alternating iterations until Nash equilibrium was achieved. The hardware configuration included 12 GB of memory, an Intel Core i5 CPU, and an NVIDIA GeForce 940M GPU.

The advantages of this technique were assessed through image restoration of the GoPro, Köhler, and Lai datasets. Peak signal-to-noise ratio (PSNR), structural similarity (SSIM), and individual parameters (Params) were used as evaluation metrics for image restoration quality. Among these, PSNR measures the difference in pixel values between reconstructed and real images, with higher values representing smaller distortion. SSIM measures the similarity between two images in terms of brightness, contrast, and structural information. Values closer to 1 indicate higher agreement between the reconstructed and real images. The parameters measured the complexity of the model, with smaller values being preferable. PSNR and SSIM are defined as follows:

(7) $$\begin{array}{c} \text{PSNR}=10\;\log\left(\frac{{\text{MAX}}^{2}}{{\text{RMSE}}^{2}}\right),\\ \text{SSIM}=\frac{\left(2\mu_{x}\mu_{y}+c_{1}\right)\left(2\sigma_{x}\sigma_{y}+c_{2}\right)\left(\sigma_{xy}+c_{3}\right)}{\left(\mu_{x}{}^{2}+\mu_{y}{}^{2}+c_{1}\right)\left(\sigma_{x}{}^{2}+\sigma_{y}{}^{2}+c_{2}\right)\left(\sigma_{x}\sigma_{y}+c_{3}\right)}, \end{array}$$

where MAX is the maximum value of image pixels (generally 255), RMSE is the root mean square error for the image, *μ*_*x*_ and *μ*_*y*_ are the mean values of images *x* and *y*, respectively, *σ*_*x*_^2^ and *σ*_*y*_^2^ are the variance of images *x* and *y*, respectively, *σ*_*xy*_ is the covariance of the two images, and *c*_1_, *c*_2_, and *c*_3_ are small constants that prevent the denominator from equaling 0. In addition, *c*_1_ = (*k*_1_ × *L*)^2^, *c*_2_ = (*k*_2_ × *L*)^2^, and *c*_3_ = *c*_2_/2, where *k*_1_ = 0.01, *k*_2_ = 0.03, and *L* = 255.

### 5.2. Simulation Analysis for the GoPro Dataset

The GoPro dataset is the highest-resolution, largest-scale, and most widely used public database for image deblurring studies [19]. This dataset provides realistic images by simulating the process of image blurring from real-time shooting. The data are comprised of 3214 pairs of blurred and sharp images with a resolution of 1280 × 720 × 3. The training set consists of 2103 pairs and the test set includes 1111 pairs. The effectiveness of the proposed model was assessed by comparing it with several mainstream algorithms applied to the same dataset. This included the traditional method of Xu et al. and deep learning methods such as those of Sun et al. and Suin et al., Deep Deblur, SRN, DeblurGAN, DeblurGAN-v2, and MPRNet. Performance metrics (PSNR, SSIM, and Params) for each algorithm are provided in Table 4.

The following are evident from these results:

1. The last column of Table 4 shows PSNR and SSIM values of 32.67 and 0.965 for the DRSN-GAN, respectively, while only 15.7 M parameters were required. This is indicative of excellent image restoration performance.
2. The DRSN-GAN, DeblurGAN [20], and DeblurGAN-v2 [21] exhibit a similar network structure, though a small full-connection network was trained to remove noise in the proposed algorithm. Compared with DeblurGAN, PSNR and SSIM increased by 14.07% and 4.1%, respectively, while ensuring a small increase in Params. Compared with DeblurGAN-v2, PSNR and SSIM increased by 10.56% and 3.32%, respectively, while Params decreased by 74.22%. These results demonstrate that the proposed method improves the quality of restored images without drastically increasing parameter quantities.
3. The study by Zamir et al. [23] is the latest published paper in the field of image deblurring. Compared with MPRNet, PSNR and SSIM were slightly higher for DRSN-GAN. In addition, Params was reduced by 21.89%, suggesting that the proposed model cannot only ensure excellent restored image quality, but can also significantly reduce parameter quantities, decrease runtime, and improve the efficiency of image restoration.

**Figure 5:**
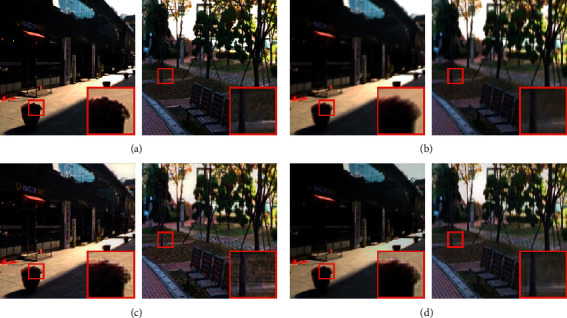
Sample (a) clear and (b) blurred images from the GoPro dataset. Corresponding results are shown for the (c) MPRNet and (d) DRSN-GAN algorithms.

The resulting image restoration effects are compared with those of MPRNet in Figure 5, where the large red frame in the lower-right corner shows an enlarged view of the small red frame for enhanced detail.

The following observations can be made:

1. Figure 5(c) indicates MPRNet enhanced the brightness of images but produced significant grid artifacts.
2. Figure 5(d) demonstrates that the proposed method achieved good results. The image was successfully restored, retaining structure and color characteristics of the original image, and the content is essentially the same. Although edge texture effects are lacking, the deblurring effect is satisfactory.
3. As can be seen in the enlarged red box in the lower-right corner of each figure, image details produced by our method are clearer and more real, and texture effects are superior.

**Figure 6:**
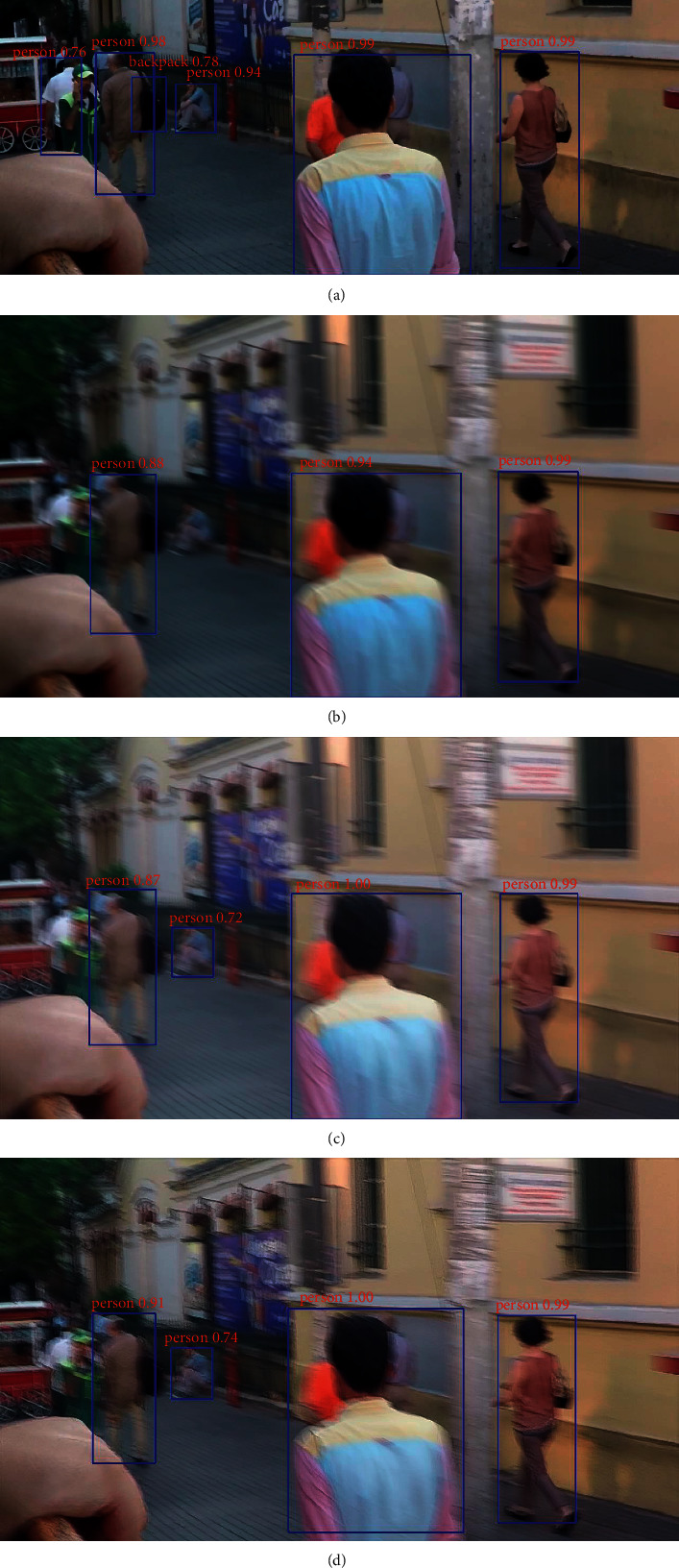
Sample (a) clear and (b) blurred images used for YOLO detection. Corresponding results are shown for the (c) MPRNet and (d) DRSN-GAN algorithms.

The YOLO detection method was also used for quantitative analysis of deblurring performance, as shown in Figure 6. In the figure, the blue line denotes the position of an object in the image and the red label in the upper-left corner indicates the object category and recognition rate. The following can be observed:

1. Figure 6(a) includes 5 people and a backpack that were identified in the clear image with a high recognition rate. In contrast, only 3 people were identified in the blurred image of Figure 6(b), with a lower recognition rate.
2. Both MPRNet and the proposed method identified four people in Figures 6(c) and 6(d). The leftmost person and the backpack were not recognized in the blurred images, while the person squatting in the middle was recognized in 6(c) and 6(d), but not in 6(b).
3. The proposed method achieved a recognition rate 0.04 higher than that of MPRNet for the leftmost person and 0.02 higher for the left-middle person. The resulting model restoration effect was also superior to that of MPRNet.

### 5.3. Simulation and Analysis for the Köhler Dataset

The Köhler dataset consists of four images, each blurred by 12 different kernels. The data were generated by recording and analyzing real camera motion, replayed on a robotic platform. A series of clear images were then recorded using a 6D camera motion trajectory, the benchmark for evaluating blind deblurring algorithms [50]. However, the images in this dataset have no detectable targets, so YOLO detection cannot be performed. The proposed technique was also applied to these data and compared with five conventional algorithms (Deep Deblur, SRN, DeblurGAN, DeblurGAN-v2, and MPRNet) using PSNR and SSIM as evaluation metrics (see Table 5).

These experimental results indicate the restoration quality produced by DRSN-GAN is superior to that of MPRNet, with PSNR and SSIM values increased by 0.01 and 0.002, respectively. Due to the influence of photographic equipment and atmospheric effects, a certain amount of noise will be superimposed on images. DRSN-GAN can remove motion blurring and noise simultaneously, with high generalizability. However, MPRNet can only perform one task at a time, such as deblurring, denoising, or removing rain.

**Figure 7:**
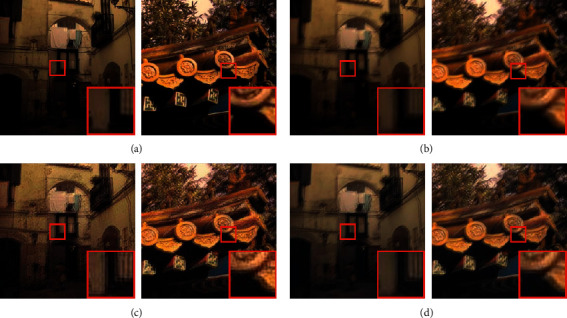
Sample (a) clear and (b) blurred images from the Köhler dataset. Corresponding results are shown for the (c) MPRNet and (d) DRSN-GAN algorithms.

The effectiveness of our algorithm for the Köhler dataset is demonstrated by the boxed area in Figure 7, from which the following can be concluded:

1. The deblurring results produced by MPRNet, shown in Figure 7(c), exhibit unnatural visual sensory effects, such as oversharpening, grid artifacts, and edge distortion.
2. Figure 7(d) suggests that, compared with the representative MPRNet model, improving the generator module eliminates obvious chromatic aberrations and color distortion. As a result, the reconstructed image is more natural and lifelike.
3. Detailed information marked by boxes in the figure indicates our method produced a more natural restoration result, particularly for fine structures.

### 5.4. Simulation and Analysis for the Lai Dataset

**Figure 8:**
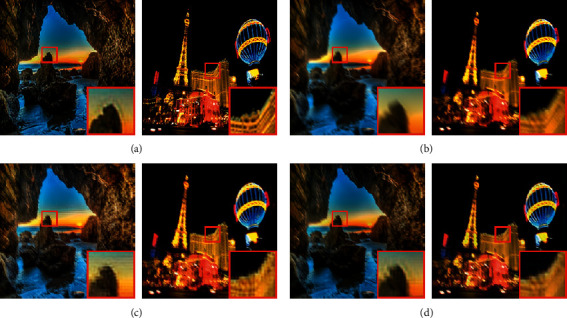
Sample (a) clear and (b) blurred images from the Lai dataset. Corresponding results are shown for the (c) MPRNet and (d) DRSN-GAN algorithms.

Lai et al. introduced a synthetic dataset that includes clear and blurred images [51] and consists of a uniform motion blur subset and a nonuniform motion blur subset. Because this paper focuses on the nonuniform motion deblurring problem, the nonuniform motion blur subset is selected as the test set. Unlike for the other two datasets, PSNR and SSIM cannot be calculated for the Lai data and YOLO detection could not be achieved. However, since the blur kernel is derived from a 6D camera trajectory, the blurred images can be acquired by rotation, translation, and scaling. As such, the images are not aligned in the usual way. In other words, there is no pixel-to-pixel correspondence between the two images. Test results from the Lai dataset were visualized to provide a comparison of the algorithms, as shown in Figure 8. The following conclusion can be drawn from these results:

1. Figure 8(c) indicates the deblurring effects produced by MPRNet for the Lai data were inferior to those of the GoPro dataset. In addition, the restored image resolution is low and the deblurring effects are generally unsatisfactory.
2. Figure 8(d) indicates the deblurring effects produced by the proposed model (for the Lai data) were also inferior to results from the GoPro dataset. However, the generated samples are relatively clearer than those of the MPRNet method and the degree of restoration is higher.
3. The red magnified box in Figures 8(c) and 8(d) demonstrates that deblurring effects produced by the proposed model were slightly better for scene details than those of the MPRNet method.

### 5.5. Simulation and Analysis for Noisy Images

**Figure 9:**
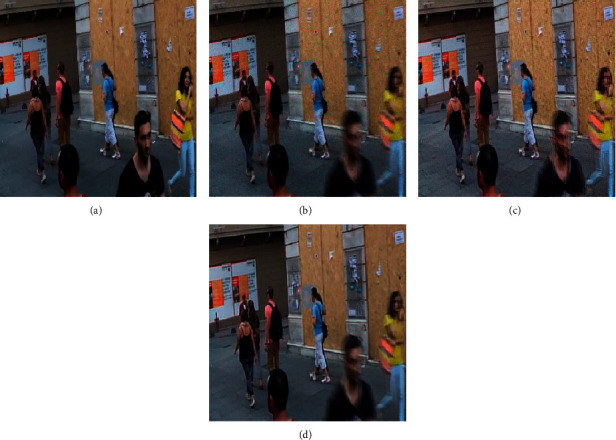
Sample (a) clear and (b) blurred and noisy images from the GoPro dataset. Corresponding results are shown for the (c) MPRNet and (d) DRSN-GAN algorithms.

**Figure 10:**
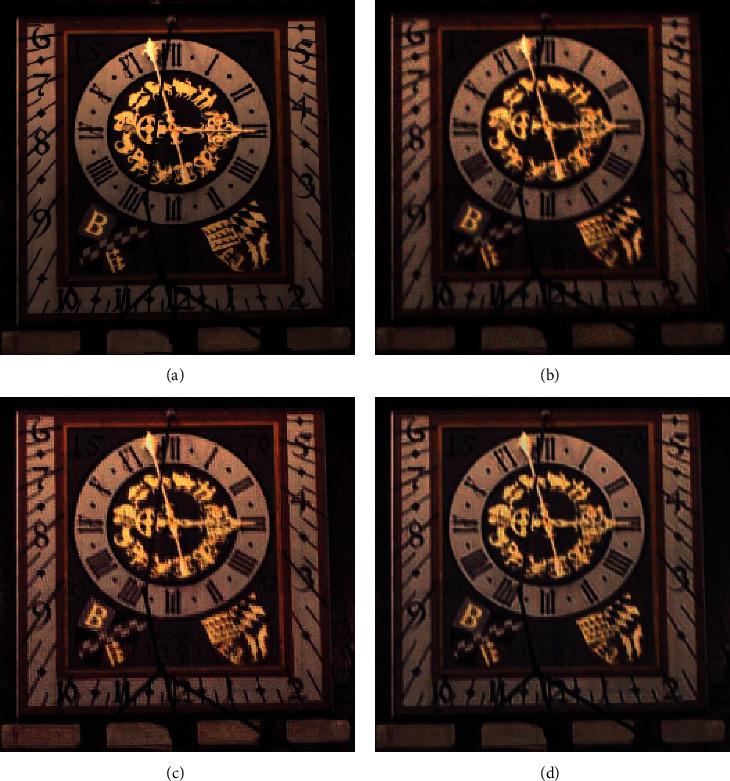
Sample (a) clear and (b) blurred and noisy images from the Köhler dataset. Corresponding results are shown for the (c) MPRNet and (d) DRSN-GAN algorithms.

The generalizability and deblurring effects of the proposed model have been demonstrated above. Gaussian noise (mean of 0, variance of 0.001) was added to images in the GoPro and Köhler datasets to illustrate the restoration abilities of the proposed method. Test results were visualized to provide a comparison of various algorithms, as shown in Figures 9 and 10. The following conclusions can be drawn from these results:

1. Figures 9(c), 9(d), 10(c), and 10(d) indicate that deblurring by DRSN-GAN was inferior to that of MPRNet.
2. Figures 9(c) and 10(c) retained significant noise, while Figures 9(d) and 10(d) are mostly noiseless.
3. The corresponding PSNR index also indicates that DRSN-GAN outperformed MPRNet.

### 5.6. Analysis and Summary

Test results from the GoPro, Köhler, and Lai databases led to the following conclusions:

1. Our proposed method offers fewer parameters and higher PSNR and SSIM values than conventional methods, which also produced unexpected effects for high-noise motion blur images.
2. The performance of the proposed algorithm across multiple datasets suggests that the model offers good generalizability while producing high-quality recovery results across multiple datasets.
3. Compared with MPRNet, the visual clarity and image quality metrics improved with application of the proposed technique, particularly for edge features and fine-scale information. The number of required parameters was also reduced by 21.89%. Unlike conventional DeblurGAN, our model includes an attention module to remove noise from blurred images. This mechanism is comprised of a small fully connected network, as shown in Figure 2. As the complexity of the network structure increased, parameter quantities increased only slightly. This demonstrates that the constructed network model and corresponding loss functions produced superior restoration effects while reducing parameter requirements.

## 6. Conclusions

A single-image restoration algorithm based on deep residual shrinkage and generative adversarial networks (DRSN-GAN) was proposed in this paper. The presented algorithm can remove noise and improve model generalizability using an attention mechanism and a soft threshold function, thereby avoiding related problems such as obvious grid effects in restored images and poor image restoration. Model training speed was also improved by changing the DRSN network structure. An end-to-end method was also applied, which avoided ringing effects and simplified algorithm steps. Experimental results demonstrated that, compared with popular algorithms such as Deep Deblur, SRN, DeblurGAN, DeblurGAN-v2, and MPRNet, the DRSN-GAN method achieved the best deblurring performance and the highest generalizability for the GoPro, Köhler, and Lai datasets. In addition, when compared with the MPRNet method, parameter quantities for DRSN-GAN were reduced significantly (21.89%). Further improving operational efficiency for the DRSN-GAN method will be the focus of future research, with an emphasis on simplifying the generator network structure and optimizing related parameters. Algorithm parameters will also be reduced further to improve PSNR and SSIM indicators. The results of this study are applicable to the restoration of high-noise images in several fields, including facial recognition, video surveillance, medical image recognition, and space exploration.

## Figures and Tables

**Table 1:** Comparison of common GAN models.

Model	Mechanisms	Advantages	Disadvantages	Applicable scenarios
GAN	Generator and discriminator	Generate high-resolution images	Training instability, mode collapse, vanishing gradient	Generating partial images
CGAN	Add additional information to the input layer of the generator and discriminator	Effectively constrain the overly free GAN	Requires labeled data, training imbalance, the quality of generated images is low	Generating specified target images
DCGAN	Combine GAN with CNN, use BN and other techniques to train the model	Rich variety of generated images	The quality of generated images is low and model training is unstable	Generating most images
WGAN	Use Wasserstein distance instead of JS divergence in traditional GAN	Prevents GAN training instability and mode collapse	Unreasonable parameter settings can easily lead to gradient dispersion	The GAN model does not converge and the mode collapses
LSGAN	Use the least squares loss function instead of a traditional cross entropy loss function	Generate high-quality samples	The gradient vanishes or explodes during training	Generating high-quality images
BigGAN	Expand the scale of the model, use truncation and orthogonal regularization to train	Model training is stable and can generate highly clear images	Large number of parameters and difficult to train	Suitable for generating highly clear images

**Table 2:** The attention mechanism algorithm.

*Step 1.* Calculate the absolute value of the result after convolution.
*Step 2.* Obtain the feature A through global mean pooling of the input after calculating the absolute value.
*Step 3.* Input the feature map after global mean pooling into a small fully connected network with a sigmoid function serving as the last layer. Normalize the output between 0 and 1 to obtain the coefficient *α.*
*Step 4.* Express the threshold as *α* × *A* and use soft threshold processing to remove redundant information and noise from the input, after the absolute value is acquired in Step 1.

**Table 3:** The DRSN-GAN algorithm.

*Step 1.* The training dataset is selected and preprocessed. The nearest neighbor image interpolation method is used to transform the resolution of the GoPro images IB from 1280 × 720 to 256 × 256.
*Step 2.* The generated image is obtained by using the generator composed of DRSN.
*Step 3.* Input the generated image and its corresponding clear image into the discriminator to obtain the probability that the generated image belongs to the clear image.
*Step 4.* Train the discriminator with the probability obtained in Step 3 and judge whether the number of times of discriminator training reaches the preset number (five times). If yes, execute Step 5; otherwise, return to Step 3.
*Step 5.* The probability of the generated image belonging to the clear image is obtained by using the trained discriminator.
*Step 6.* Determine whether the generator and the discriminator reach Nash equilibrium. If so, execute Step 8; otherwise, execute Step 7.
*Step 7.* Train the generator with the probability obtained in Step 5, reset the number of times of discriminator training to zero, and return to Step 2
*Step 8.* Use the trained generation network to remove image motion blur.

**Table 4:** A comparison of PSNR, SSIM, and Params for various algorithms using the GoPro dataset.

Method	PSNR	SSIM	Params (M)
Xu et al. [9]	25.10	0.890	—
Sun et al. [16]	24.64	0.842	—
Deep Deblur [19]	29.23	0.916	11.8
SRN [49]	30.10	0.932	6.8
DeblurGAN [20]	28.64	0.927	14.5
DeblurGAN-v2 [21]	29.55	0.934	60.9
Suin et al. [22]	31.85	0.948	23.1
MPRNet [23]	32.66	0.959	20.1
DRSN-GAN	32.67	0.965	15.7

**Table 5:** A comparison of PSNR and SSIM values for various algorithms using the Köhler dataset.

	Sun et al. [16]	Deep Deblur [19]	SRN [49]	DeblurGAN [20]	DeblurGAN-v2 [21]	MPRNet [23]	DRSN-GAN
PSNR	25.22	26.48	26.75	26.10	26.72	26.80	**26.81**
SSIM	0.773	0.807	0.837	0.816	0.836	0.836	**0.838**

## Data Availability

The data used to support the findings of this study are available from the corresponding author upon request.
